# Symptoms of Selective Mutism in Non-clinical 3- to 6-Year-Old Children: Relations With Social Anxiety, Autistic Features, and Behavioral Inhibition

**DOI:** 10.3389/fpsyg.2021.669907

**Published:** 2021-05-31

**Authors:** Peter Muris, Nona Monait, Lotte Weijsters, Thomas H. Ollendick

**Affiliations:** ^1^Department of Clinical Psychological Science, Maastricht University, Maastricht, Netherlands; ^2^Departement of Sielkunde, Stellenbosch University, Stellenbosch, South Africa; ^3^Department of Psychology, Virginia Polytechnic and State University, Blacksburg, VA, United States; ^4^Department of Psychology, Roehampton University, London, United Kingdom

**Keywords:** selective mutism, social anxiety, autism spectrum disorder, behavioral inhibition, children

## Abstract

Selective mutism (SM) is a psychiatric condition that is characterized by a failure to speak in specific social situations (e. g., at school) despite speaking normally in other situations (e.g., at home). There is abundant evidence that anxiety, and social anxiety in particular, is a prominent feature of SM, which is the main reason why this condition is currently classified as an anxiety disorder. Meanwhile, there is increasing support for the notion that autism-related problems are also involved in SM. The present study examined the relations between SM and social anxiety, autistic features, and behavioral inhibition to the unfamiliar (i.e., the tendency to react with restraint and withdrawal when confronted with unfamiliar stimuli and situations). Parents of 172 3- to 6-year-old preschool children completed an online survey for measuring the relevant constructs. Results showed that there were positive and statistically significant correlations between SM and social anxiety, autistic features, and behavioral inhibition. Regression analyses revealed that (1) both social anxiety and autistic features accounted for a significant and unique proportion of the variance in SM scores, and (2) that both of these variables no longer made a significant contribution once behavioral inhibition was added to the model. It can be concluded that while the involvement of social anxiety is unambiguous in SM, autism-related problems are also implicated. Furthermore, behavioral inhibition seems to play a key role in the non-speaking behavior of non-clinical young children.

## Introduction

The prototypical feature of children with selective mutism (SM) is a total absence of speech in specific social situations (e.g., school) while showing a normal ability to speak in other situations [e.g., at home; American Psychiatric Association, 2013]. Thus, children with this condition often remain consistently silent in the classroom and do not respond verbally to questions and invitations of the teacher or to verbal and non-verbal communication attempts of their classmates. However, when at home with their parents, siblings, other family members, or friends, these children speak and communicate normally, expressing themselves verbally just like their peers. SM is a psychiatric disorder that usually becomes manifest during the early school years before children reach the age of 5 years (Steinhausen and Juzi, 1996). Children showing SM in its extreme form (i.e., <1%; Bergman et al., 2002; Karakaya et al., 2008) are usually referred to a clinical facility for treatment because the persistent non-speaking behavior obviously hinders them in performing adequately in school and establishing friendships with other children (Manassis, 2009). It is good to bear in mind, however, that SM is a dimensional phenomenon as there are also young children who are not totally silent in certain social situations, but clearly use less spoken language as they would do in other settings (Gensthaler et al., 2020). This means that SM, just like other psychiatric conditions, is better conceptualized as a continuum rather than as a categorical diagnostic entity, and this justifies that its scientific inquiry should not only be conducted in clinical samples but also in non-clinical populations.

When looking at the origin of the selective non-speaking behavior that is so characteristic for children with SM, it is now generally assumed that social anxiety plays an important role. The scientific evidence for this notion comes from various sources. To begin with, children with SM frequently have a comorbid diagnosis of social anxiety disorder (SAD; e.g., Black and Uhde, 1995; Yeganeh et al., 2003; Vecchio and Kearney, 2005). More precisely, in a meta-analysis by Driessen et al. (2020), structured clinical interview data of 837 children with SM that were pooled from 22 studies indicated that SM was frequently accompanied by a co-occurring anxiety disorder, and in the vast majority of cases (i.e., 69%) SAD was implicated. Furthermore, Vogel et al. (2019) conducted qualitative interviews in 65 children with SM aged 8 to 18 years to explore the content of their fears in speech-related situations. It was found that the fears of children with SM were primarily focused on themes that are also typical for children with SAD, such as the fear of being negatively and critically evaluated by other people. Finally, comparisons of the symptom picture between children with SM and children with SAD have shown many similarities in the clinical presentation of both disorders (Manassis et al., 2003; Yeganeh et al., 2006; Gensthaler et al., 2016b; Milic et al., 2020). Most importantly, it has been noted that SM and SAD are difficult to distinguish on behavioral, psychophysiological, self-, parent-, and teacher-report measures of social anxiety (Poole et al., 2020). Given all these research findings, the current classification of SM as an anxiety disorder (see American Psychiatric Association, 2013) seems justified (Sharp et al., 2007; Viana et al., 2009; Muris and Ollendick, 2015), with some scholars even pleading for the recognition of SM as a special variant of SAD (Bögels et al., 2010).

In a recent review paper, Muris and Ollendick (2021b) suggested that—besides social anxiety—autism-related problems might also be implicated in SM. This point-of-view is controversial as autism spectrum disorder (ASD) is generally considered as an exclusion criterion for SM (American Psychiatric Association, 2013). In its purest form, the non-speaking behavior of children with SM and children with ASD is quite different. More specifically, children with SM display the prototypical muteness when exposed to socially demanding situations, but in comfortable situations their interaction with other people is normal, with a full range of emotion and adequate social cognition (Thom et al., 2020). In contrast, children with ASD display a more generalized impairment in social interaction: in both comfortable and uncomfortable circumstances, they exhibit impediments in social emotion, cognition, skills, and motivation (Pallathra et al., 2018), that may sometimes be expressed in non-speaking behavior. However, as noted above, contemporary psychiatry views mental disorders as dimensions with low symptom levels on the one hand and high symptom levels on the other hand (Krueger and Piasecki, 2002; Hudziak et al., 2007), which implies that relations among disorders and overlap in symptoms are the rule rather than the exception (Plana-Ripoll et al., 2019).

Indeed, there is increasing evidence showing that SM and ASD are related psychopathological conditions and that it is difficult to maintain an absolute diagnostic boundary between both disorders. For instance, in a study by Steffenburg et al. (2018), the medical records of 97 children with SM were subjected to a systematic analysis to establish the possible presence of ASD. The results showed that no less than 63% of the children with SM also fulfilled the diagnostic criteria for ASD, while an additional 20% of the children with SM displayed autistic features, hence showing subclinical signs of this neurodevelopmental disorder. Although the Steffenburg et al. (2018) study has some methodological shortcomings (e.g., reliance on retrospective chart reviews), findings do suggest that there is considerable co-occurrence of SM and ASD. In a further investigation, Klein et al. (2019) administered a standardized parent- and teacher-report scale to assess psychopathological symptoms in 42 children with SM aged between 2 and 14 years. The scale also contained a screen for ASD and it was found that 80% of the children with SM scored above the cut-off on this autism probability index, indicating that many of them showed clinical signs of social and communication problems and stereotyped interests and behaviors. In addition, Cholemkery et al. (2014) asked parents of 6- to 18-year-old children with SM, social anxiety disorder, and ASD as well as typically developing children to complete a standardized scale measuring autistic symptoms in five domains, namely social awareness, social cognition, social communication, social motivations, and repetitive/restricted behaviors. The results showed that all children with a clinical diagnosis displayed higher levels of autistic symptoms than the typically developing children. Children with ASD clearly displayed the highest levels of social interaction impairments, but on two domains (i.e., social communication and social motivation) children with SM also exhibited elevated scores (as compared to children with social anxiety disorder), which implies that they were also relatively high on the autism spectrum. Other research has indicated that children with SM appear to display a similar cognitive deficit (i.e., impairments in initiating joint attention; Nowakowski et al., 2011) and share a common genetic liability (i.e., a specific polymorphism in the contactin-associated protein-like 2 gen; Stein et al., 2011) as young people with ASD.

In view of this increasing evidence, Muris and Ollendick (2021b) argued that the presence of ASD (or at least autistic traits) likely increases children's proneness to develop SM. In specific, the social skills and social cognition deficits associated with this neurodevelopmental problem might fuel social anxiety symptoms as well as prompt muteness as an avoidance strategy to deal with the excessive symptomatology elicited by specific social situations. Further, the rigidity and cognitive inflexibility of children with ASD will enhance social difficulties thereby further intensifying the social anxiety, but also promoting the persistent non-speaking behavior displayed by children with SM. It is important to note that in most of children, SM is primarily an anxiety-driven condition (Cohan et al., 2008; e.g., Capozzi et al., 2018). However, various scholars have noted out that SM is a heterogeneous disorder (e.g., (Mulligan, 2012)) and that there are children in which—besides (social) anxiety—other problems such as developmental delay (Kristensen, 2000), language expression difficulties (Manassis et al., 2007), oppositional behavior (Diliberto and Kearney, 2018), and presumably ASD or related features are implicated as well.

Temperament might be another factor contributing to SM. Of special interest is the temperament typology of “behavioral inhibition to the unfamiliar” (BIU; Kagan, 1997), which can be defined as a predisposition characterized by restraint in engaging with the external world combined with a tendency to search the environment for potential threats and to avoid or withdraw from unfamiliar people and situations. A host of studies have established that BIU is an important risk factor for SAD (Clauss and Blackford, 2012), but given the fact that reduced speech in social situations is one of the defining features of an inhibited temperament (e.g., Garcia Coll et al., 1984; Van Brakel et al., 2004), it is obvious to also explore its link with SM. In a first study of this topic, Gensthaler et al. (2016a) employed a retrospective parent-rating scale to measure inhibited temperament features in 3- to 18-year-old children with SM, SAD, other internalizing problems, and healthy controls. It was found that children with SM and SAD were reported to have been more inhibited during their early childhood years than children with other internalizing behaviors and healthy controls. In general, the levels of BIU of children with SM and ASD were rated as comparably high, although on the specific domain of shyness children with SM even displayed higher levels of inhibition than their counterparts with SAD. Further research by Milic et al. (2020) relied on a cross-sectional, multi-method research design to compare BIU features among children with SM, children with SAD, and non-clinical controls. Parent ratings revealed that children with SM and children with SAD both had a greater tendency to withdraw from novel situations and unfamiliar people than the non-clinical control children. Observations conducted during a series of performance tasks indicated that children with SM scored higher on a few measures of inhibition (i.e., latency to initiate gestures, latency to initiate speech, total amount of speech) than children with SAD. In a final investigation by Muris et al. (2016), 57 non-clinical children aged 3 to 6 years performed two speech tasks to assess the number of spoken words, while their parents completed a set of questionnaires for measuring children's levels of SM, social anxiety, and an inhibited temperament. Significant associations were noted among all variables, but the correlation between BIU and SM symptoms was particularly robust, and it was also found that this temperament typology was the best predictor of the number of spoken words during the standardized speech tasks. Taken together, the available evidence demonstrates that BI, which already has been established as an important risk factor for SAD, is also clearly implicated in SM.

The observation that SM is associated with multiple factors fits nicely within a developmental psychopathology framework (Cicchetti and Cohen, 2006). That is, the selective non-speaking behavior of children with SM does not seem to develop as the result of one deterministic variable, but likely originates from a complex of vulnerability factors that jointly increase the probability (risk) for this psychiatric condition to occur (Cohan et al., 2006; Viana et al., 2009; Muris and Ollendick, 2015). In keeping with the principle of equifinality (i.e., any one outcome might result from multiple and diverse pathways; see Cicchetti and Rogosch, 1996), the exact constellation of vulnerability factors can be and most likely is different across children. However, it is important to study the relative contributions of various risks on a group level as such information can be highly relevant for giving direction to the clinical management of young people with a given disorder.

So far, the research on etiological models of SM has primarily examined vulnerability factors in isolation. The evidence suggests that social anxiety plays a dominant role in the origins of this disorder, but other variables such as autistic features and a behaviorally inhibited temperament also seem to be implicated. Meanwhile, we know little about the unique contributions of each of these variables to SM. This seems all the more important when acknowledging that there appears to be considerable overlap among these vulnerability factors. More precisely, SAD and ASD appear to be closely related (Spain et al., 2018) and the same is true for BIU and SAD (Ollendick and Hirshfeld-Becker, 2002; Clauss and Blackford, 2012). So far, little is known about the link between BIU and ASD, although it should be noted that children with autism-related problems often display reticence and distress when meeting unfamiliar people or facing novel situations, which is also typical for temperamental inhibition (Ersoy, 2019).

With these issues in mind, the present study made a first attempt to examine the (unique) relations between social anxiety, autistic features, and BIU on the one hand and symptoms of SM on the other hand. For this purpose, the parents of 172 non-clinical children aged 3 to 6 years completed a survey containing the Selective Mutism Questionnaire (SMQ; Bergman et al., 2008), the social anxiety subscale of the Preschool Anxiety Scale-Revised (Edwards et al., 2010), the Autism Spectrum Questionnaire (ASQ; Van der Ploeg and Scholte, 2014), and the Behavioral Inhibition Questionnaire-Short Form (BIQ-SF; Edwards, 2007). It was hypothesized that there would be positive correlations between SM and the other constructs. The most substantial associations were expected to be found between SM symptoms and social anxiety/BIU, whereas the relation between SM symptoms and autistic features was expected to be considerably smaller. Furthermore, based on theoretical notions (Muris and Ollendick, in press), it was hypothesized that even when controlling for social anxiety, autistic features will still make a unique contribution to SM symptoms. The role of BIU was investigated more exploratively, but on the basis of an earlier study (Muris et al., 2016) it can be expected that this temperament typology makes a significant contribution to symptoms of SM even when controlling for its shared variance with the other constructs and social anxiety in particular.

## Method

### Participants and Procedure

Participants in this study were the parents of 172 non-clinical children (96 boys and 76 girls) aged 3 (*n* = 45, 26.2%), 4 (*n* = 61, 35.5%), 5 (*n* = 42, 24.4%), or 6 (*n* = 24, 14.0%) years; the mean age was 4.26 years (*SD* = 1.00). The sample was recruited via 3 daycare facilities and two elementary schools in the Southern part of The Netherlands, as well as by means of a snowball sampling method (Goodman, 1961) starting with the acquaintances of the second and third author using online social media platform. To be included in the study, participants needed to be the parent of a child in the preschool age range (3 to 6 years) and to possess sufficient command of the Dutch language in order to be able to complete the questions of the survey. There were no exclusion criteria for this study.

In most cases, the mothers completed the survey (*n* = 152, 88.3%). All families had a Caucasian background and the vast majority of the parents and children (*n* = 169, 98.2%) were of Dutch nationality; only some families included members with South European or Middle Eastern roots. The language spoken at home was mainly Dutch (*n* = 146, 84.9%) or the Dutch dialect that is typically spoken in this part of The Netherlands (*n* = 24, 13.9%); a foreign language (Italian and Greek) was the dominant language in only two families (1.2%).

Parents first received an information letter describing the purpose of this study and an informed consent form. After signing the informed consent form, they were sent a link guiding them to the online survey. Following this, parents completed the set of questionnaires describing their child's behaviors in relation to the relevant constructs. After finishing the questionnaires, the parents were given the opportunity to share their email address in case they wished to receive information on the results of the study. The study was approved by the Ethical Review Committee of Psychology and Neuroscience at Maastricht University (reference number: ERCPN-221_50_03_2020).

### Assessment

Symptoms of SM were measured with the *SMQ* (Bergman et al., 2008), which is a 17-item parent-rating scale measuring the frequency of non-speaking behavior in three settings where children are normally expected to speak: at school (e.g., “When appropriate, my child speaks in groups or in front of the class”), at home/with family (e.g., “When appropriate, my child speaks with family friends who are well-known to him/her”), and other social situations (e.g., “When appropriate, my child speaks to store clerks and/or waiters”). Items were rated on a 4-point Likert scale, with 0 = never, 1 = seldom, 2 = often, and 3 = always. A total score (range 0–51) can be calculated by summing ratings across all items. Lower scores on the SMQ indicate a lower frequency of speaking behavior and thus higher levels of SM. To enhance interpretability, the main analyses were conducted using a reversed SMQ total score for which higher scores reflect higher symptom levels of SM. Previous studies have shown that the SMQ is a reliable scale (with Cronbach's in the 0.80 to 0.90 range) that relates in a theoretical meaningful way with other measures (Bergman et al., 2008; Letamendi et al., 2008), predicts the diagnostic status of SM (Oerbeck et al., 2020), and is sensitive to document treatment effects (Bergman et al., 2013; Oerbeck et al., 2015).

A subscale of the *PAS-R* (Edwards et al., 2010) was used to measure children's level of social anxiety. The PAS-R is a 30-item adaptation of the Preschool Anxiety Scale (Spence et al., 2001), a parent-report questionnaire that assesses symptoms of anxiety disorders in young children. The social anxiety subscale consists of 6 items such as “My child worries that he/she will do something to look stupid in front of other people,” and “My child is afraid to go up to a group of children to join their activities.” Items are scored on a 4-point Likert scale ranging 0 = not at all true to 4 = very often true. A total social anxiety score (range 0–28) can be computed, with higher scores indicating higher levels of social anxiety symptomatology. In general, the PAS-R has been shown to be a reliable and valid index of anxiety in children of a preschool age. The internal consistency and test-retest reliability estimates of the social anxiety scale are in the 0.70 to 0.80 range, its scores are predictive of a clinical diagnosis of SAD, and correlate significantly and robustly with other measures of anxiety and emotional symptoms (Edwards et al., 2010; Stuijfzand and Dodd, 2017; Orgiles et al., 2018; Gudmundsdottir et al., 2019).

The *ASQ* (Van der Ploeg and Scholte, 2014) evaluates the presence of symptoms of ASD in children. The questionnaire consists of 24 items that can be allocated to two subscales: (1) Interactive and communicative problems (all reversed items, e.g., “My child actively seeks contact with other children,” “My child gets along with different kinds of people”), which covers the persistent social interaction and social communication impairments displayed by young people with this neurodevelopmental condition, and (2) Odd, deviant behaviors (e.g., “My child shows strange, repetitive behaviors,” “My child has difficulties when he/she has to switch from one task to another”), which pertains to the restricted repetitive behaviors and interests of children with ASD. Parents rate the applicability of each item for their child using a 5-point Likert-type scale ranging from 1 = not at all to 5 = very much. Scores can be computed for the full scale as well as for the two subscales by summing the ratings across relevant items. The ratings on items referring to Interactive and communicative problems are recoded, so that in all cases higher scores reflect higher levels of ASD symptomatology. Psychometric evaluation of the ASQ (Van der Ploeg and Scholte, 2014) has indicated that the scale is reliable in terms of internal consistency (with Cronbach's alphas ranging between 0.91 and 0.94 for non-clinical children and between 0.84 and 0.90 for children with ASD) and test-retest stability (with intraclass correlations over a 4-week period varying between 0.84 and 0.91) as well as interrater agreement (intraclass correlations between 0.62 and 0.82). Further, scores on the ASQ discriminate well between children with and without ASD and correlate positively and substantially with an alternative measure of symptoms of this neurodevelopmental disorder.

The *BIQ-SF* (Edwards, 2007) is the short version of the Behavioral Inhibition Questionnaire (Bishop et al., 2003). This parent-report scale contains 14 items measuring features of the temperament typology of BIU in children. Representative items include “My child gets upset when being left in new situations for the first time, for example kindergarten” and “My child approaches new situations or activities very hesitantly,” which parents rate on a 6-point Likert scale, ranging from 1 = hardly ever to 6 = almost always. A total score can be computed by summing ratings across all items, with a higher score being indicative for a higher level of BIU. The internal consistency of the BIQ-SF was demonstrated to be good (with a Cronbach's alpha of 0.92 for the total score) and scores on the scale were found to be fairly stable over a period of 1 to 2 years (test-retest correlations being 0.73 and 0.65, respectively; Vreeke et al., 2012). In addition, support was obtained for the validity of the BIQ-SF as scores correlated positively and significantly with behavioral observations of young children's inhibited temperament (Bishop et al., 2003; Vreeke et al., 2012).

### Data Analyses

**Figure 1:**
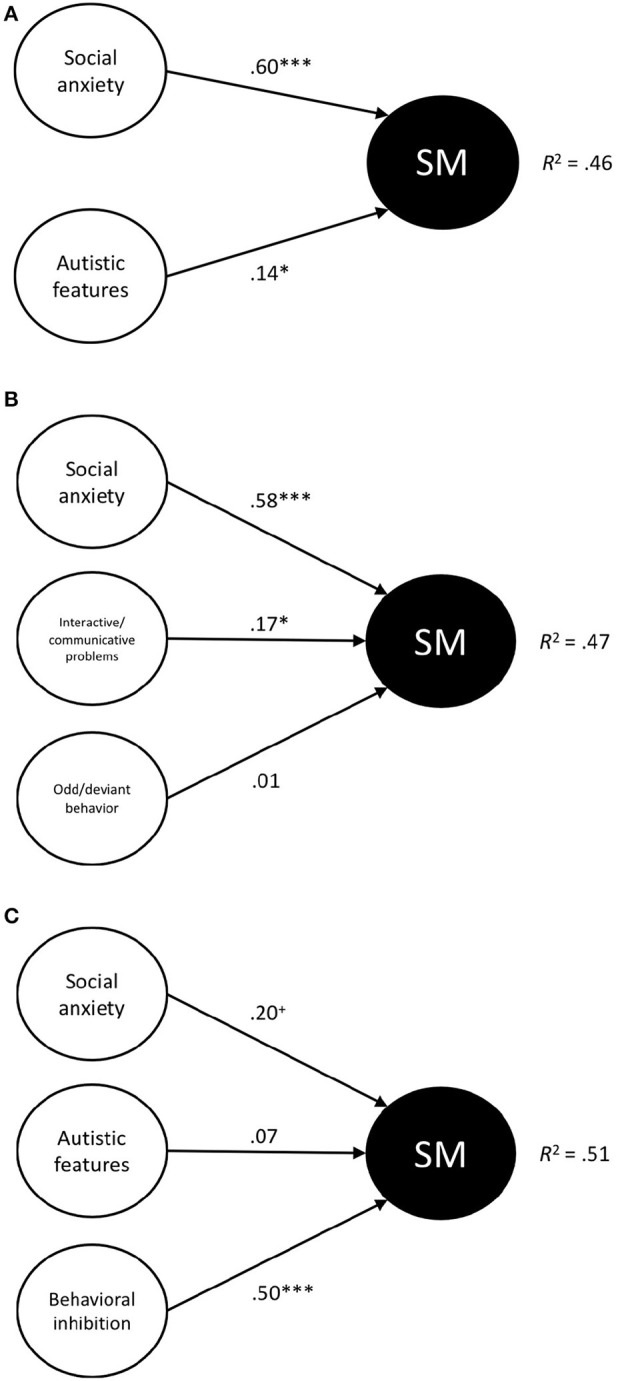
**(A–C)** Schematic representation of the three regression models that were tested. Standardized betas and *R*^2^ values are shown. SM, Symptoms of Selective Mutism. ^+^*p* < 0.10, *p < 0.05, ***p < 0.001.

Data were analyzed using the Statistical Package for Social Sciences (SPSS Version 25). First, descriptive statistics (mean scores, standard deviations, reliability coefficients) were calculated, and gender differences and age effects were investigated by means of independent *t*-tests and correlations, respectively. Second, Pearson correlations were computed to examine the relations between symptoms of SM (SMQ) on the one hand and social anxiety (PAS-R), autistic features (ASQ), and BIU (BIQ-SF) on the other hand. Third, to explore unique contributions of various constructs to symptoms of SM, linear regression analyses were conducted with the SMQ total score was the dependent variables and other constructs served as the predictors. In specific, three models were tested (see Figure 1). In a first model, it was explored whether social anxiety (PAS-R) and autistic features (ASQ total score) each explain a unique proportion of the variance in selective mutism symptoms (SMQ), which would be in line with notion of Muris and Ollendick (in press). The second model was basically a refinement of the first model. Apart from the social anxiety (PAS-R), the two subscales of the ASQ were included separately in the regression equation in order to find out the relative contributions of Interactive/communicative problems and odd/deviant behaviors. The third and final model not only included social anxiety and autistic features but also incorporated behavioral inhibition as predictor, and hence will give insight on the relative contributions of psychopathology indicators and temperament characteristics to SM symptoms in non-clinical children.

## Results

### General Findings

Before addressing the main results of the present study, a number of general findings are reported. First, a comparison of the SMQ, PAS-R, ASQ, and BIQ-SF scores with normative data of these measures (Spence et al., 2001; Vreeke et al., 2012; Van der Ploeg and Scholte, 2014; Oerbeck et al., 2020) revealed that parents rated the children in the present study as clearly falling in the normal range of selective mutism, social anxiety, autistic features, and behavioral inhibition. Second, demographic variables did not have a significant influence on the constructs that were assessed in this study. That is, no gender differences were found for any of the measures (all *t*'s ≤ 1.54, *p*'s ≥ 0.11), implying that boys and girls were rated as displaying comparable levels of psychopathology and temperament. Further, no significant relationships were noted between age and the assessed constructs (*r*'s between −0.08 and 0.11, *p*'s ≥ 0.16), which was not that surprising given that the age range of the children included in the present study was quite small (i.e., 3 to 6 years). Third and finally, all questionnaires showed good to excellent internal consistency reliability, with Cronbach's alpha coefficients varying between 0.81 and 0.94 (see Table 1).

**Table 1:** Mean scores (standard deviations) and reliability coefficients for parent-report questionnaires of children's symptoms and temperament features as well as Pearson correlations among these measures.

	***M* (*SD*)**	**Cronbach's α**	**1**	**2**	**3**	**4**	**5**
1. SMQ Selective mutism	38.36 (10.07)^†^	0.93					
2. PAS-R Social anxiety	9.51 (5.62)	0.90	0.67^***^				
3. BIQ-SF Behavioral inhibition	41.36 (14.64)	0.94	0.71^***^	0.88^***^			
4. ASQ Autistic features	52.72 (11.56)	0.89	0.43^***^	0.48^***^	0.54^***^		
5. ASQ Interactive/communicative problems	26.61 (6.83)	0.87	0.47^***^	0.52^***^	0.60^***^	0.89^***^	
6. ASQ Odd/deviant behaviors	26.11 (6.27)	0.81	0.28^***^	0.31^***^	0.33^***^	0.87^***^	0.56^***^

*N = 172. SMQ, Selective Mutism Questionnaire; PAS-R, Preschool Anxiety Scale-Revised; BIQ-SF, Behavioral Inhibition Questionnaire-Short Form; ASQ, Autism Spectrum Questionnaire*.

† *Mean and standard deviation as calculated for the original SMQ total score. For the purpose of the correlation analysis, the SMQ total score was reversed so that higher scores indicated higher levels of SM symptoms*.

*** *p < 0.001*.

### Correlations Between SM, Social Anxiety, Autistic Features, and BIU

Correlations between SM symptoms and symptoms of social anxiety and ASD as well as features of the temperamental construct BIU are shown in Table 1. Three main conclusions can be derived from this table. First, a robust positive and substantially significant correlation was found between the SMQ and the PAS-R social anxiety subscale (*r* = 0.67): as hypothesized, higher levels of SM symptoms were accompanied by higher levels of social anxiety. Second, substantial positive and statistically significant correlations were also noted between BIQ-SF scores on the one hand and SMQ and PAS-R social anxiety scores on the other hand (*r*'s being 0.71 and 0.88, respectively), which means that higher levels of the temperament characteristic of BIU were associated with higher levels of both SM and social anxiety. Third, SMQ scores were also positively correlated with ASQ scores (*r* = 0.43), which means that symptoms of SM were associated with higher levels of autistic features. A test for comparing correlated correlation coefficients indicated, as predicted, that the correlation between SM and social anxiety was significantly stronger than the correlation between SM and ASD features (*Z* = 3.97, *p* < 0.001).

An additional finding that emerged from the correlational analysis was that SMQ scores were statistically significantly correlated with both the interactive/communicative problems and odd/deviant behaviors subscales of the ASQ [*r*'s being 0.47 and 0.28, with the former correlation being significantly stronger than the latter correlation (*Z* = 2.91, *p* < 0.01)].

Further, a statistically significant positive correlation was found between PAS-R social anxiety and ASQ scores (*r*'s between 0.31 and 0.52), which indicates that higher levels of social anxiety symptoms were accompanied by higher levels of autistic features. A final result concerned the positive and statistically significant correlations between BIQ-SF and ASQ scores: notably, higher levels of BIU were associated with higher levels of autistic features (*r* = 0.54) and this appeared true for interactive/communicative problems (*r* = 0.60) as well as odd/deviant behaviors (*r* = 0.33).

### Unique Contributions of Various Constructs to Symptoms of SM

To examine the unique contributions of various constructs to symptoms of SM, three linear regression analyses with SMQ scores as the dependent variable were conducted (see above: section Data Analyses). As demographic did not have a large impact on the variables included in this study, we did not include them as predictors in the regression models. However, running the analyses with gender and age as additional predictors yielded highly similar results. Before discussing the results of the regression analysis, two general remarks should be made. First, diagnostic tests were conducted to detect multicollinearity issues. Results showed that Variance Inflation Factor values were all ≤ 4.84, while Tolerance values were ≥ 0.22, which points out that there were no substantial violations of multicollinearity (O'Brien, 2007). Second, it was found that each of the tested models were statistically significant (all *F*'s ≥ 49.00, *p*'s < 0.001) and that across various analyses predictor variables explained between 46 and 51% of the total variance in symptoms of SM.

The main results of the three regression analyses are shown in Table 2. The first regression analysis with the PAS-R social anxiety score and ASQ total score as the predictors revealed that both social anxiety (β = 0.60, *t* = 9.32, *p* = 0.000) and autistic features (β = 0.14, *t* = 2.22, *p* = 0.028) made a positive, statistically significant, and independent contribution to symptoms of SM. The second analysis in which PAS-R social anxiety and ASQ subscales were the predictor variables confirmed the unique contribution of social anxiety (β = 0.58, *t* = 8.76, *p* = 0.000) and also showed that the effect of autistic features was mainly carried by the interactive/communicative difficulties associated with this neurodevelopmental condition (β = 0.17, *t* = 2.18, *p* = 0.031). No statistically significant effect of odd/deviant behaviors was found (β = 0.01, *t* = 0.09, *p* = 0.930). The third and final regression analysis included PAS-R social anxiety, the ASQ total score, and BIQ-SF as predictors, and found that only the temperament trait of BIU made a unique and statistically significant contribution to symptoms of SM (β = 0.50, *t* = 4.23, *p* = 0.000). In this model, the contribution of social anxiety was also positive but only marginally significant (β = 0.20, *t* = 1.73, *p* = 0.086), whereas ASD symptoms did no longer explain a significant proportion of the variance in SM symptomatology (β = 0.07, *t* = 1.09, *p* = 0.278).

**Table 2:** Main results of the regression analyses in which symptoms of SM were predicted from social anxiety, autistic features, and behavioral inhibition.

	***B***	**95% CI**	***SE***	**β**	***R*^**2**^**
SMQ Selective mutism^†^					0.46^***^
PAS-R Social anxiety	1.07	[0.85, 1.30]	0.12	0.60^***^	
ASQ Autistic features	0.12	[0.01, 0.24]	0.06	0.14^*^	
SMQ Selective mutism					0.47^***^
PAS-R Social anxiety	1.04	[0.80, 1.27]	0.12	0.58^***^	
ASQ Interactive/communicative problems	0.24	[0.02, 0.46]	0.11	0.17*	
ASQ Odd/deviant behaviors	0.01	[−0.21, 0.23]	0.11	0.01	
SMQ Selective mutism					0.51^***^
PAS-R Social anxiety	0.35	[−0.05, 0.75]	0.20	0.20^+^	
ASQ Autistic features	0.06	[−0.05, 0.17]	0.06	0.07	
BIQ-SF Behavioral inhibition	0.34	[0.18, 0.50]	0.08	0.50^***^	

*N = 172. SMQ, Selective Mutism Questionnaire; PAS-R, Preschool Anxiety Scale-Revised; BIQ-SF, Behavioral Inhibition Questionnaire-Short Form; ASQ, Autism Spectrum Questionnaire*.

† *The SMQ total score was reversed so that higher scores indicated higher levels of SM symptoms*.

+ *p < 0.10*,

* *p < 0.05*,

*** *p < 0.001*.

## Discussion

The present study examined psychopathological and temperamental correlates of SM symptoms in a non-clinical sample of 3- to 6-year-old children by means of a parent survey. The results revealed there was a robust and statistically significant correlation between social anxiety and SM symptoms. This is in line with previous clinical studies showing that the comorbidity between SAD and SM is high (Driessen et al., 2020) and that there are clear similarities between both disorders in terms of fear content (Vogel et al., 2019) and clinical presentation (Manassis et al., 2003; Yeganeh et al., 2006; Gensthaler et al., 2016b; Milic et al., 2020; Poole et al., 2020). But even in non-clinical research, the substantial correlation between symptoms of social anxiety and SM has been documented (Muris et al., 2016). On the basis of the intimate link between both conditions it has been argued that SM can best be viewed as a special variant of SAD. Some advocates of this notion have suggested that SM should be regarded as a more extreme variant of SAD (e.g., Black and Uhde, 1992), while others have put forward that SM can best be viewed as an early developmental manifestation of SAD (e.g., Bergman et al., 2002). Importantly, the robust association with social anxiety justifies the position of SM among the anxiety disorders, which has also implications for the clinical management of the disorder (Muris and Ollendick, 2021a). More specifically, clinicians should use instruments to assess the level of social anxiety associated with this condition and apply cognitive-behavioral interventions to treat the fear-driven non-speaking behavior of children with this condition (Bergman et al., 2013; Oerbeck et al., 2014; Cornacchio et al., 2019).

Furthermore, it was found that autistic features were also positively associated with symptoms of SM. When controlling for concurrent levels of social anxiety the relation between ASD and SM symptoms clearly attenuated but still remained positive and statistically significant. This corroborates results obtained in previous clinical research showing that a substantial proportion of the children with SM display autistic features (Steffenburg et al., 2018; Klein et al., 2019) as well as cognitive or pathophysiological features associated with this neurodevelopmental disorder (Nowakowski et al., 2011; Stein et al., 2011). This result also provides further support for the model recently described by Muris and Ollendick (2021b) in which ASD-related problems are proposed as one of the psychopathological phenomena contributing to the persistent non-speaking behavior of children with SM. These authors assume that both symptom clusters of ASD, namely (a) social communication and interaction difficulties, and (b) restricted and repetitive behaviors and interests, each make an independent contribution to SM. The present results indicate that although both ASD symptoms clusters were significantly and positively correlated with symptoms of SM, only the communication and interaction difficulties (as measured by ASQ Interactive/communicative problems) made a unique statistically significant contribution. The most plausible explanation for this finding is that communication and interaction deficiencies are directly relevant for children's social functioning (Pallathra et al., 2018) and thus exert their influence even when symptom levels are relatively low. Meanwhile, repetitive and restrictive behaviors and interests are not necessarily social in nature and hence may need to be more intense and severe before they start to have an impact on children's (speaking) behavior in social situations. According to normative data of the ASQ (Van der Ploeg and Scholte, 2014), scores on both the “interactive/communicative problems” and “odd/deviant behavior” subscales were rather low in this non-clinical population (i.e., mean scores fell in the lowest decile of children with ASD). Thus, it seems important to test the relative contributions of both symptoms clusters to SM in a sample of clinically referred children who will not only show higher levels but also more variation in the prototypical symptoms of this neurodevelopmental disorder, which could result in finding that repetitive and restrictive behaviors and interests also play a role in SM (see e.g., Magiati et al., 2016; Teh et al., 2017).

This study also showed that there is a strong relationship between SM and BIU, which is in agreement with the results of previous research (Gensthaler et al., 2016a; Muris et al., 2016). In the present study, not only a robust positive correlation between SM symptoms and features of this temperament characteristic was found, but the results of the regression analysis also demonstrated that even when controlling for symptoms of social anxiety and ASD, BIU still made a significant and unique contribution to SM symptoms. In fact, BIU emerged as the only statistically significant predictor variable, while social anxiety and autistic features no longer explained a significant proportion of the variance once this temperament factor was added to the regression model. Again, this may well have to do with the non-clinical sample that was investigated in this study, in which symptom levels of social anxiety, autistic features, and SM in general were quite low. The fact that BIU appeared to display a unique relation with SM aligns with the notion of Perez-Edgar and Guyer (2014) that this temperament characteristic can be seen as a prodrome of anxiety pathology which can be easily and reliably detected in non-clinical populations.

An additional finding of the current investigation concerned the positive relation between BIU and autistic features. Surprisingly, few studies have directly examined this link, although the key features of BIU share similarities with the typical clinical symptoms of “insistence on sameness” and “reactions of distress to small changes” displayed by many children with ASD (American Psychiatric Association, 2013). One exception is a recent investigation by Esroy et al. (2020) who found some evidence that children at high risk for ASD displayed higher levels of BIU than children who were at low risk for this neurodevelopmental disorder. Meanwhile, there is also research indicating that temperament and personality features related to BIU such as high emotional instability (neuroticism), low sociability (extraversion), and low effortful control (see Muris and Dietvorst, 2006) are more clearly present in children with ASD than in typically developing children (e.g., Samyn et al., 2011; Macari et al., 2017; Lodi-Smith et al., 2019).

It needs to be acknowledged that the present investigation suffers from a number of limitations. First, as already mentioned, the study relied on a non-clinical sample of children displaying relatively low symptom levels of SM and other psychopathologies who were not subjected to a formal psychiatric evaluation. So, replication of this research in clinically referred children or non-clinical children who are carefully assessed for psychiatric disorders seems very important to gain more insight on the relations between SM on the one hand and social anxiety, autistic features, and BIU on the other hand. Second, this study solely relied on parent-report questionnaires to measure symptoms of SM and the other constructs. Because all scales included items that were concerned with the assessment of social difficulties, the data were particularly prone to the common-method variance bias. Given the young age of the children that were included in this study, the use of child self-reports was not feasible, but obviously observation-based procedures as well as the employment of scales to be completed by day care facility workers and teachers would have provided important cross-validational information. Third, an important part of the data was collected via snowball sampling, a method that was used because due the Covid-19 pandemic schools and daycare facilities were either closed or less willing to participate in research because they were already overloaded by handling other logistic issues. However, a disadvantage of the snowball sampling method is that one does not know to what extent results are generalizable to the whole population (Balter and Brunet, 2012). Fourth, although this investigation focused on a number of relevant psychopathological and temperamental correlates of SM, it is good to keep in mind that other factors have also been connected to this condition. Prominent examples are developmental delays (Kristensen, 2000), speech and language problems (Manassis et al., 2007), and externalizing symptoms (Diliberto and Kearney, 2018). Thus, in order to get a complete picture of possible antecedents of children's non-speaking behavior, it will be necessary to also include scales or instruments that assess these constructs. Fifth and finally, the study was correlational in nature, which means that although the main analyses were conducted with symptoms of SM as the to be explained (i.e., dependent) variable, in actuality no conclusions can be drawn in terms of cause-effect relationships. Thus, prospective, longitudinal research in which symptoms of SM, social anxiety, ASD and temperamental inhibition are repeatedly assessed over the course of the preschool and early primary school years could provide important information on the temporal associations among these constructs and the psychopathological and temporal antecedents of SM in children.

In spite of these shortcomings, the current findings indicate that non-speaking behavior in children is positively associated with social anxiety, autistic features, and the temperament characteristic of BIU, and thus support theoretical notions on the multifactorial origins of SM (Cohan et al., 2006; Viana et al., 2009; Muris and Ollendick, 2015, 2021b). In this sample of non-clinical children, BIU appeared to be the best “predictor” of SM symptoms, which suggests that this temperament trait might be a particularly important target for prevention strategies. In this light, it is good to note that Rapee and Edwards (2009) have developed a parent-based intervention programs by means of which inhibited behaviors in children can be effectively reduced. The program has already been shown to be effective in reducing the development of common childhood anxiety disorders such as separation anxiety disorder and social phobia (Rapee et al., 2010), and so it would be of interest to determine if this approach would also be successful in preventing the development of SM.

## Data Availability Statement

The raw data supporting the conclusions of this article will be made available by the authors, without undue reservation.

## Ethics Statement

The studies involving human participants were reviewed and approved by Ethical Research Committee of Psychology and Neuroscience, Maastricht University. Written informed consent to participate in this study was provided by the participants' legal guardian.

## Author Contributions

PM designed the study, supervised the data collection and processing, conducted the statistical analyses, and wrote the article. NM and LW were involved in the practical implementation of the study: they designed the web survey, collected, and processed the data. TO contributed to the theoretical foundation of the study and assisted in writing the article. All authors contributed to the article and approved the submitted version.

## Conflict of Interest

The authors declare that the research was conducted in the absence of any commercial or financial relationships that could be construed as a potential conflict of interest.
